# Component gap control during posterior-stabilised total knee arthroplasty using the posterior condylar pre-cut technique

**DOI:** 10.1186/s40634-021-00398-z

**Published:** 2021-09-15

**Authors:** Makoto Kawasaki, Ryutaku Kaneyama, Hitoshi Suzuki, Teruaki Fujitani, Manabu Tsukamoto, Ken Sabanai, Toru Yoshioka, Nobukazu Okimoto, Ryuji Nagamine, Akinori Sakai

**Affiliations:** 1 Department of Orthopaedic Surgery, School of Medicine, University of Occupational and Environmental Health, 1-1 Iseigaoka Yahatanishi-ku, Kitakyushu City, Fukuoka 807-8555 Japan; 2 Knee Joint Reconstruction Center, Shonan Kamakura General Hospital, 1370-1 Okamoto, Kamakura City, Kanagawa 247-8533 Japan; 3 Department of Orthopaedics, Shimura Hospital, 3-13 Funairimachi Naka-ku, Hiroshima, 730-0841 Japan; 4 Okimoto Clinic, 185-4 Yutakamachikubi, Kure City, Hiroshima 734-0304 Japan; 5 Center of Artificial Joint and Rheumatism, Fukuoka Tokushukai Medical Center, 4-5 Sugukita, Kasuga City, Fukuoka 816-0864 Japan

**Keywords:** Pre-cut trial, Gap control, Posterior stabilised TKA, Extension gap, Flexion gap, Bone gap, Component gap

## Abstract

**Purpose:**

Adjusting the gap lengths to ensure equal lengths in both extension and flexion during total knee arthroplasty (TKA) is important for achieving successful outcomes. We designed a new pre-cut trial component (PCT) for posterior-stabilised (PS) TKA and aimed to determine whether the pre-cut technique is useful for component gap (CG) control in PS TKA.

**Methods:**

A total of 70 knees were included. The PS PCT for PS TKA is composed of a 9-mm-thick distal part and 5-mm-thick posterior part with a cam structure. First, the distal femur and proximal tibia were cut to create an extension gap. Next, a 4-mm pre-cut was made from the posterior femoral condylar line; then, the PS PCT was attached, and the CGs were checked and compared at 0° and 90° knee flexion. Final CGs with the trial femoral components were compared with gaps in PS PCT at 0° and 90° knee flexion.

**Results:**

CGs using PS PCTs were 10.2 mm at 0° and 13.6 mm at 90° knee flexion. According to the release of the posterior capsule at intercondylar notch and the adjustment of the cutting level of posterior femoral condyle, the final CG on knee extension was 11.3 mm; it did not significantly differ from CGs with PS PCT. The final CG at 90° knee flexion was 12.7 mm; it did not significantly differ from the estimated gap (12.4 mm) in PS PCT after flexion gap control.

**Conclusion:**

CG control using PS PCT is a useful technique during PS TKA.

**Level of evidence:**

Level IV: Case series.

## Introduction

During total knee arthroplasty (TKA), adjusting the gap lengths to be equal in both extension and flexion is an important factor for achieving successful outcomes [2, 4]. The restoration of equal extension and flexion gaps is a widely accepted surgical goal of TKA as it reduces the incidence of stiffness [1] and instability [23]. In TKA, these spaces are estimated as the extension and flexion gaps after bone resection. However, gaps without the trial femoral component (bone gaps; BGs) after bone resection often differ from those after setting the trial femoral components (component gaps; CGs). The intraoperative CG difference is larger than the estimated CG difference from the BG; thus, there is a significant decrease regarding knee extension, not flexion, after femoral component placement [2, 16]. The decrease in CGs during extension may be due to tension on the posterior capsule, affected by the condyles of the femoral component [2, 16].

Minoda et al. suggested that the BGs in flexion should be reduced in size component with the BGs in extension before implantation to decrease CG differences between knee extension and flexion [15]. Onodera et al. demonstrated that excess posterior femoral condylar offset relative to the posterior wall of the tibia in knee extension (posterior offset ratio) differs in each TKA implant model, and the posterior protrusion of the posterior offset of the femoral component has a risk of flexion contracture after implantation [21]. However, using the measured resection and modified gap-balancing techniques, the CG can only be assessed after completing bone resection and setting the trial femoral component.

Therefore, preparation for setting the femoral component using a “pre-cut trial component” (PCT) before the final cutting of the posterior femoral condyle is useful [7]. However, the usual pre-cut technique is only used for cruciate-retaining (CR) TKA. Thus, we designed a novel PS PCT to reproduce the cam structure for posterior-stabilised (PS) TKA. The cam structure occupies a large portion of the intercondylar notch and tenses the soft tissues around the intercondylar notch, which may affect the CGs. Hence, the purpose of this study was to clarify whether the cam structure of PS TKA affects the CGs and whether the pre-cut technique could be useful for PS TKA.

## Methods

Between January 2016 and June 2018, one surgeon performed PS TKA (Persona® The Personalized Knee; Zimmer Biomet Holdings, Inc., Warsaw, IN, USA) on 87 knees using pre-cut methods at our institution. This case series was approved by the institutional review board of our institution (approval No. H27-048). Informed consent was obtained from all patients before the study. Over a 1-year follow-up period, 70 knees (63 patients; 12 men, 51 women) with a femorotibial angle (FTA) of over 175° varus osteoarthritis (OA) who underwent primary PS TKA were enrolled. The exclusion criteria were knees with rheumatoid arthritis (eight knees), valgus OA (four knees), less than a year follow-up (one knee), without the release of the femoral intercondylar notch capsule (one knee) and insufficient intraoperative gap measurement data (three knees), i.e. a total of 17 knees, were excluded. Age at the time of surgery was 74 ± 7.4 (mean ± standard deviation; range, 57–91 years). Preoperative FTA was 183.5° ± 4.1° (range, 175º–199º). Preoperative range of motion (ROM) using lateral X-ray evaluation was − 5.6° ± 5.9° (range, − 18°–5°) in extension and 117° ± 13.9° (range, 83°–150°) in flexion. Postoperative ROM using lateral X-ray evaluation at 1-year follow-up was 0.69° ± 5.0° (range, − 19° –13°) in extension and 120° ± 12.2° (range, 73° –144°) in flexion.

### PS pre-cut trial component

**Fig. 1:**
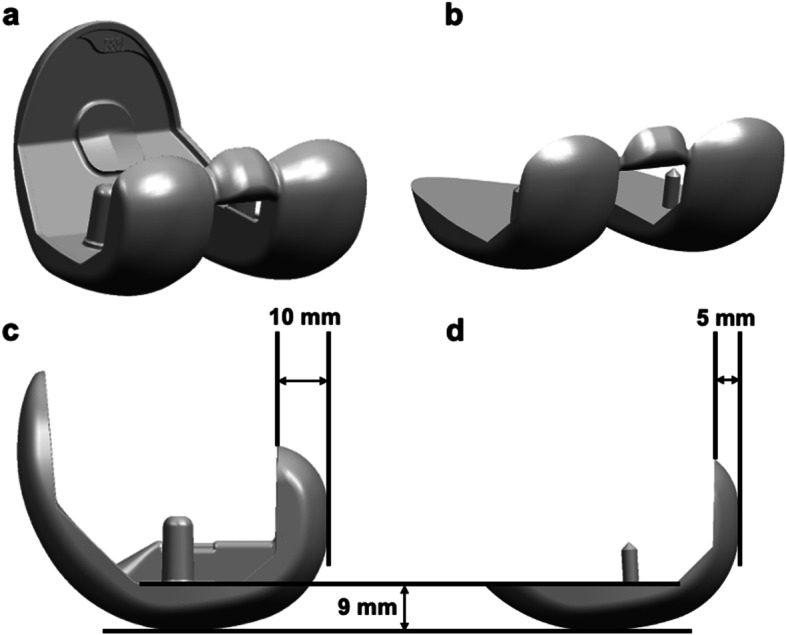
Femoral component and posterior-stabilised (PS) pre-cut trial (PCT). The femoral component (**a**) and PS PCT (**b**). PS PCT has the same superficial shape as the femoral component. The femoral component has a 9- and 10-mm thickness in the distal and posterior parts, respectively (**c**); PS PCT has 9- and 5-mm thickness in the distal and posterior parts, respectively (**d**)

**Fig. 2:**
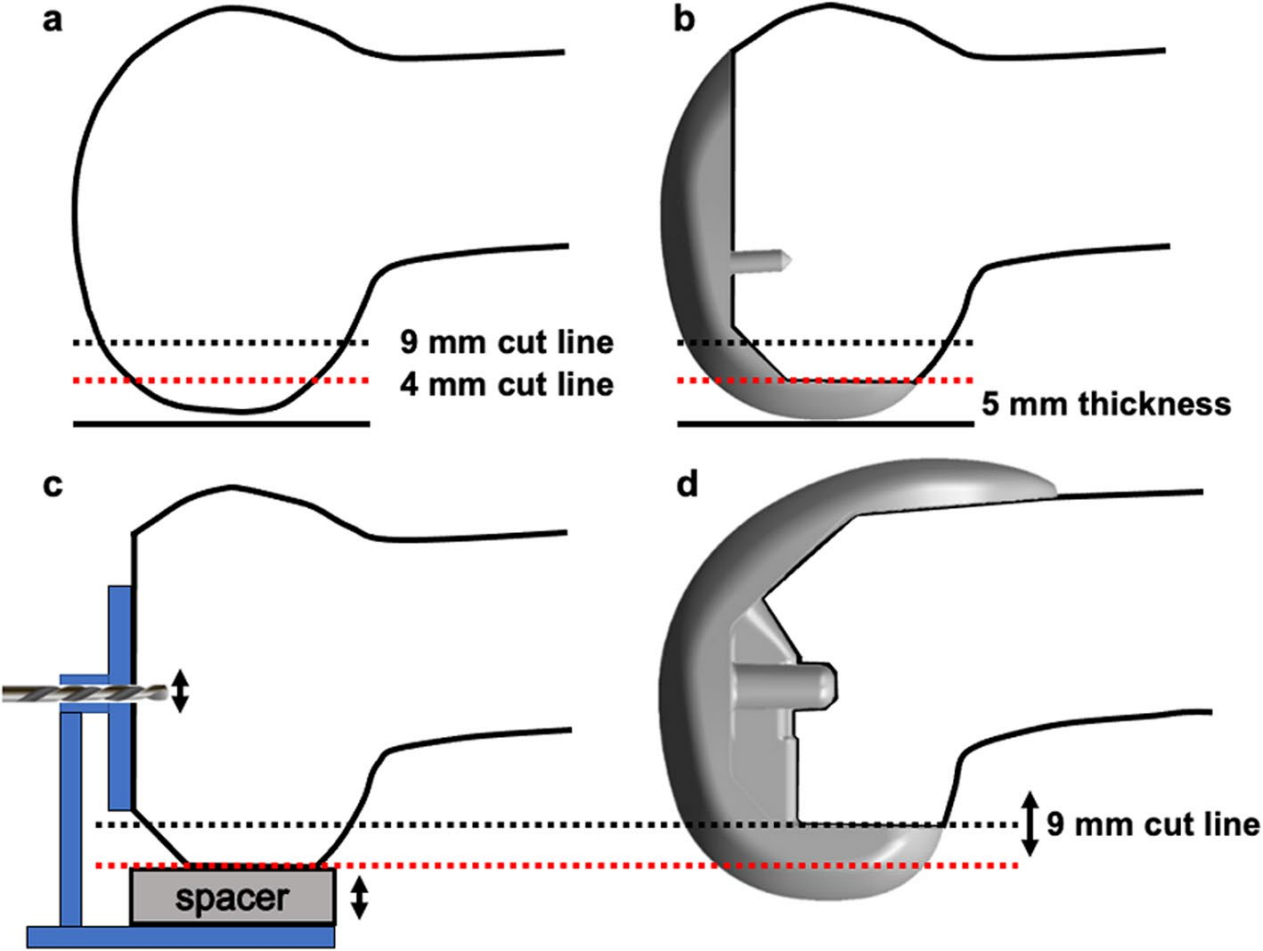
Procedure from the initial bone cutting to setting the final component. First, a 4-mm pre-cut (red dotted line) was performed (**a**). Second, PS PCT was set on the pre-cut surface (**b**). According to the gap measurement, after adjusting the cutting line of the posterior condyle posteriorly or anteriorly, a drill hole is made to install the cutting device (**c**). When the flexion gap was larger (or smaller) than the extension gap, a surgeon could control the additional cutting line (black dotted line) posteriorly (or anteriorly) in 1-mm increments using a thicker (or thinner) spacer (**c**). Lastly, the femoral trial component was set after flexion gap control (**d**). PS PCT, posterior-stabilised pre-cut trial component

**Fig. 3:**
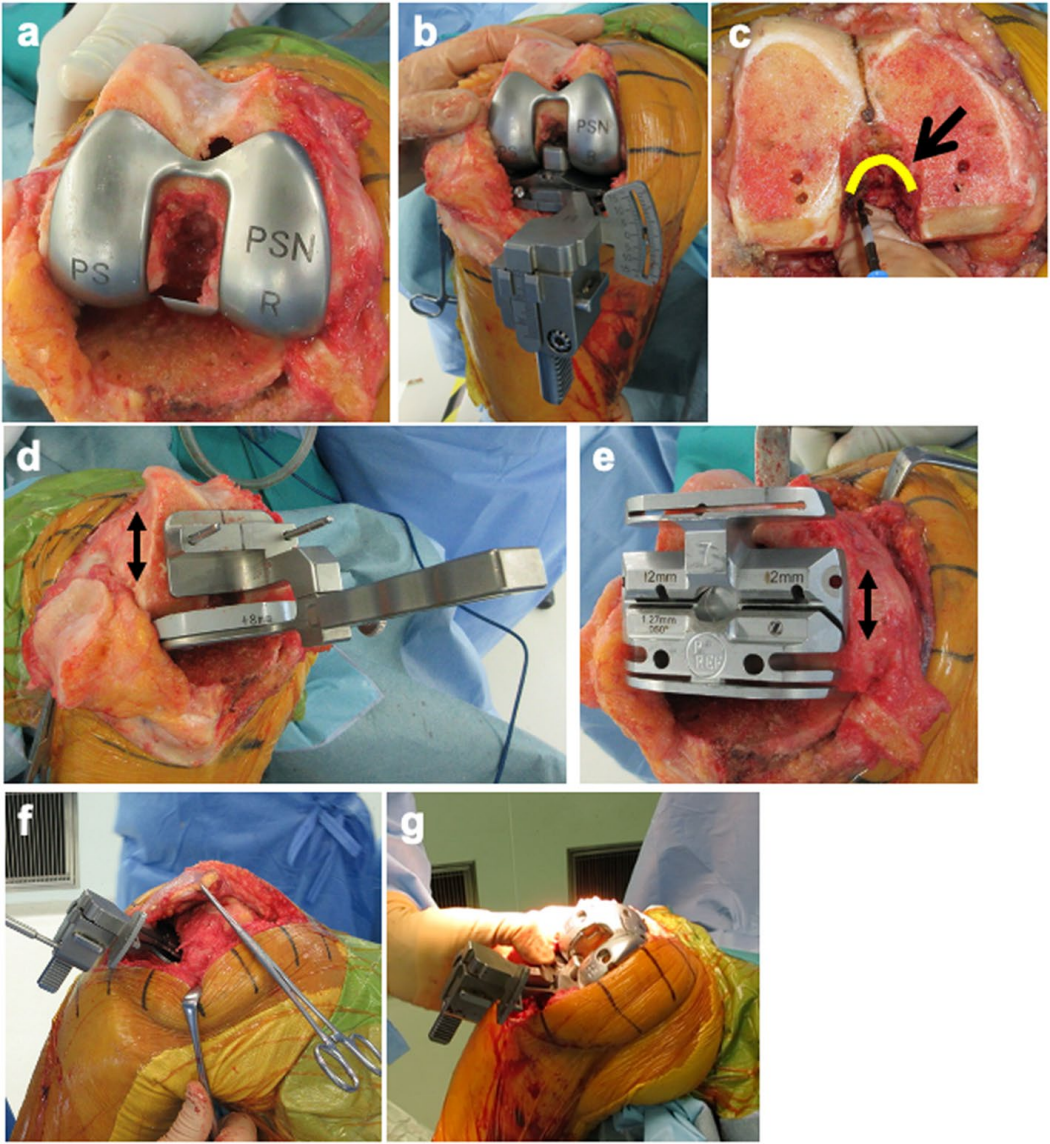
Surgical procedures. The PS PCT was attached to the femur (**a**), and the component gaps (CGs) by PS PCT were assessed at knee extension and flexion using the Offset Repo-Tensor® (OFR tensor) (**b**). Release around the intercondylar notch was performed by electrosurgical knife (**c**). In cases in which the flexion gaps were larger than the extension gaps, a small amount of resection of the posterior femoral condyle (less than component thickness; additional resection of < 5 mm) was performed to decrease the flexion gap by moving the drill hole posteriorly (**d**). The final cutting device was placed in the drill hole (**e**). After final bone resection, bone gaps (BGs) were measured (**f**), and final CGs with the trial femoral components after creation of the intercondylar box for post-cam structure were measured at knee extension and flexion (**g**). PS PCT, posterior-stabilised pre-cut trial component

The femoral component of the Persona® PS TKA system is composed of a 9-mm-thick distal part and 10-mm-thick posterior part (Fig. 1a and c). The PS PCT for PS TKA has the same superficial shape as the femoral component; however it is composed of a 9-mm-thick distal part and 5-mm-thick posterior part with a cam structure and lacks the anterior part of the femoral component (Fig. 1b and d). Compared with the PCT for CR TKA [7], the PS PCT has a cam structure in the femoral intercondylar notch space and a 1-mm-thicker posterior structure than CR PCT, to avoid reducing the strength of the cam structure. A pre-cut guide was used to produce a 4-mm pre-cut (Fig. 2a) on the lateral posterior femoral condyle [7]. After setting a PS PCT with a 5-mm-thick posterior part of the femur, it was similar to the 10-mm-thick femoral component setting when using the measured resection technique (Fig. 2b). According to the gap measurement, after adjusting the cutting line of the posterior condyle posteriorly or anteriorly, a drill hole was made to install the cutting device (Fig. 2c). When the flexion gap was larger (or smaller) than the extension gap, the surgeon could control the cutting line posteriorly (or anteriorly), in 1-mm increments, using a thick (or a thin) spacer (Fig. 2c). The final cutting device was placed in the drill hole (Fig. 3e). The final trial component after creation of the intercondylar box for the post-cam structure was set on the femur (Fig. 2d).

### Operative procedures and gap measurements

Medial parapatellar arthrotomy was performed. First, the anterior (ACL) and posterior cruciate ligaments (PCL) were resected. The distal femur and proximal tibia were cut perpendicularly to the mechanical axis of the lower leg. After extension gap creation, the femorotibial joint was distracted with a force of 40 lbs (18.1 kg) using the Offset Repo-Tensor® (OFR tensor; Zimmer Biomet Holdings) [11, 12, 16]. Joint gap angles were measured between the femoral and tibial cut surfaces in extension, and between the posterior condylar line and tibial cut surface in 90° flexion; distraction force was loaded several times until the joint centre gap remained stable, to reduce the error that may result from soft-tissue creep as previously described [11, 12, 16].

The rotation of the femoral component was set at 3°–6° to the femoral posterior condylar line, with reference to the femoral surgical transepicondylar axis (sTEA) by preoperative CT. Next, a 4-mm pre-cut was made from the lateral posterior condylar line of the femoral posterior condyle for use with the PS PCT of the femur. Once all osteophytes were removed, the PS PCT was attached to the femur (Fig. 3a) and the CGs with PS PCT were assessed at 0° and 90° knee flexion using the OFR tensor (Fig. 3b).

In all cases, except one excluded cases, the extension CGs with PS PCT became narrow and the femoral intercondylar notch capsule needed release. Release of the intercondylar notch capsule was performed as described previously [19] (Fig. 3c). The influence of the PS PCT cam structure was examined to determine whether release of the intercondylar notch capsule affected extension CGs. In order for the final CGs for extension and flexion to be equal, in cases wherein the flexion gaps were larger than the extension gaps because of the PCL resection, a small amount of resection of the posterior femoral condyle (less than component thickness; additional resection of < 5 mm) was performed to decrease the flexion gap by moving the drill hole posteriorly (Fig. 3d). The final cutting device was placed in the drill hole (Fig. 3e). After the final bone resection, we measured the BGs at 0° and 90° knee flexion (Fig. 3f). Then, the intercondylar box for the post-cam structure was created before setting the trial femoral components. Final CGs with the usual trial femoral components were compared with gaps in PS PCT at 0° and 90° knee flexion (Fig. 3g). Further, the CGs estimated from the BG (BG minus 9 mm) were compared with the final CGs.

### Clinical outcomes

The Japanese Orthopaedic Association (JOA) scores for the four domains of pain on walking, pain on ascending or descending stairs, ROM, and joint effusion for OA knees were used as the clinical outcomes in the preoperative and postoperative (at least 1 year after surgery) periods [3, 18, 20].

### Statistical analysis

All data are presented as mean ± standard deviation. Statistical analyses were performed using one-way ANOVA followed by Tukey–Kramer multiple comparison tests when the other three groups were compared, and paired t-tests when two groups were used to compare the pre-cut CGs with the final CGs. The relationship between clinical outcomes and the gap difference in the final extension and flexion gaps was analysed using regression analysis. All statistical analyses were performed using JMP version 14.2 (SAS Institute Inc., Cary, NC, USA). Statistical significance was set at *P*-value of < 0.01.

## Results

### Changing extension gaps with PS PCT and trial components

**Fig. 4:**
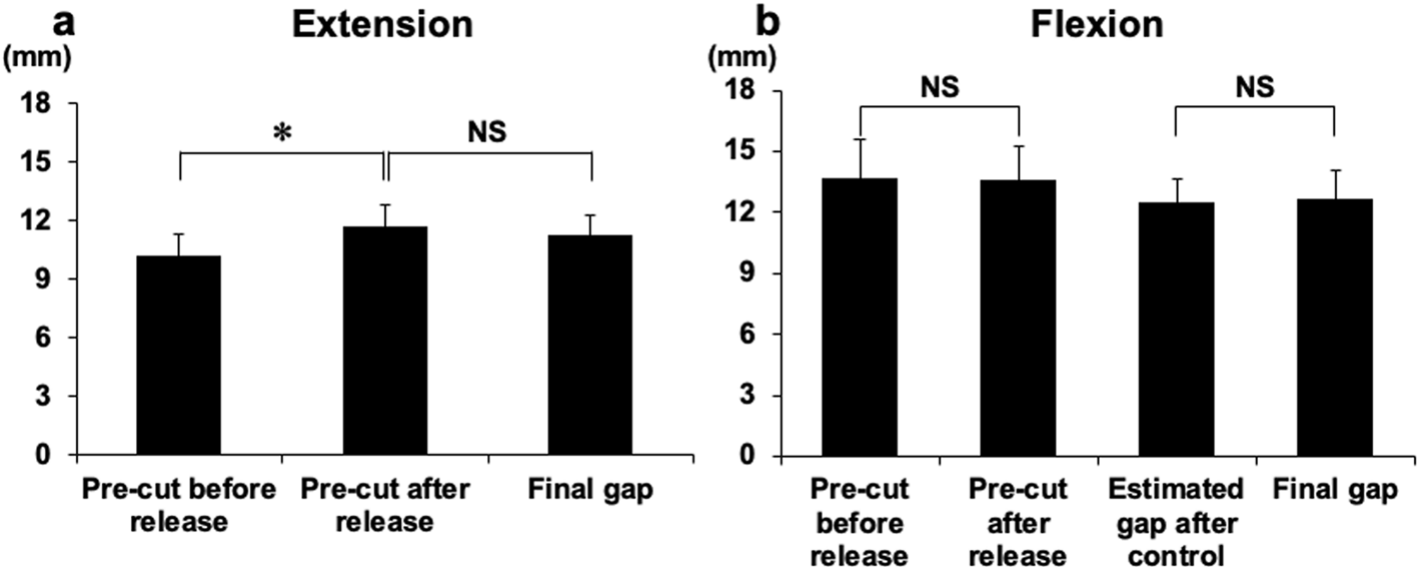
Changing of extension and flexion gaps with PS PCT and trial components. The component gaps (CGs) with PS PCT before and after the release of femoral intercondylar notch capsule, and the final CGs with femoral trial component were examined at extension (**a**). The CGs at flexion with PS PCT before and after the capsular release were also examined; then, the estimated final CGs after gap control (flexion CGs with PS PCT minus the gap control amount) and actual final CGs at flexion were compared (**b**). (*P < 0.01). PS PCT, posterior-stabilised pre-cut trial component

The CGs at extension with PS PCT before and after the release of posterior capsule at intercondylar notch release were 10.1 ± 1.2 and 11.6 ± 1.2 mm, respectively. The release of the posterior capsule at the femoral intercondylar notch was performed in all cases; the extension gaps significantly increased by 1.5 ± 0.73 mm, compared with those before release in PS PCT. The final CG with femoral trial at extension was 11.3 ± 1.1 mm. The final CGs were not significantly altered, compared with those after release in the PS PCT (Fig. 4a).

### Changing flexion gaps with PS PCT and trial components

The CGs at 90° knee flexion with PS PCT before and after the posterior capsular release were 13.7 ± 2.0 and 13.6 ± 1.7 mm, respectively. Intercondylar notch release of the posterior capsule did not significantly change the flexion CGs. An additional cut after a 4-mm pre-cut of the posterior femoral condyle was performed as follows: 5 mm (not posteriorly of cutting level; total 9-mm cut) in 18 knees, 4 mm (1 mm posteriorly of cutting level; total 8-mm cut) in 27 knees, 3 mm (2 mm posteriorly of cutting level; total 7-mm cut) in 20 knees, and 2 mm (3 mm posteriorly cutting level; total 6-mm cut) in 4 knees. In any of the cases, no cut was ≥ 6 mm (1 mm anterior to the cutting level; total 10-mm cut) and < 1 mm (4 mm posteriorly of cutting level; total 5-mm cut). The average gap control amount was 1.1 ± 0.87 mm posterior to the cutting level. The estimated final CGs in flexion (flexion CGs with PS PCT minus the gap control amount were 12.4 ± 1.2 mm) were not significantly different from the final CGs with trial component after gap control (12.7 ± 1.4 mm) (Fig. 4B).

### Difference between the estimated CGs from BGs, actual CGs with PS PCT and trial component at knee extension and flexion

**Fig. 5:**
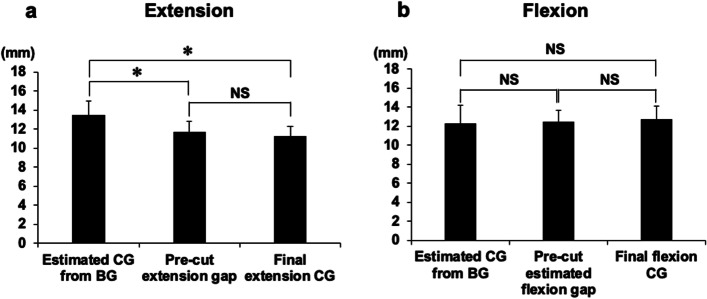
Difference between BGs and CGs. Indicated is the difference between the estimated component gaps (CGs) from bone gaps (BGs), and the actual CGs with PS PCT and trial component at knee extension and flexion. The CGs estimated from BGs at knee extension (BG minus 9 mm) (**a**) and flexion (BG minus 10 mm) (**b**) were compared with the CGs with PS PCT and actual final CGs. (*P < 0.01). PS PCT, posterior-stabilised pre-cut trial component

The CGs estimated from BGs at knee extension (BG minus 9 mm) and flexion (BG minus 10 mm) were 13.5 ± 1.5 and 12.2 ± 2.0 mm, respectively. The CGs estimated from the BGs at knee extension were significantly larger than the CGs with PS PCT and final CGs (Fig. 5a and b). The final CGs were 2.2 ± 1.1 mm smaller than the CGs estimated from BGs at knee extension.

### Relationship between the gap control amount and the final CGs at knee extension

**Fig. 6:**
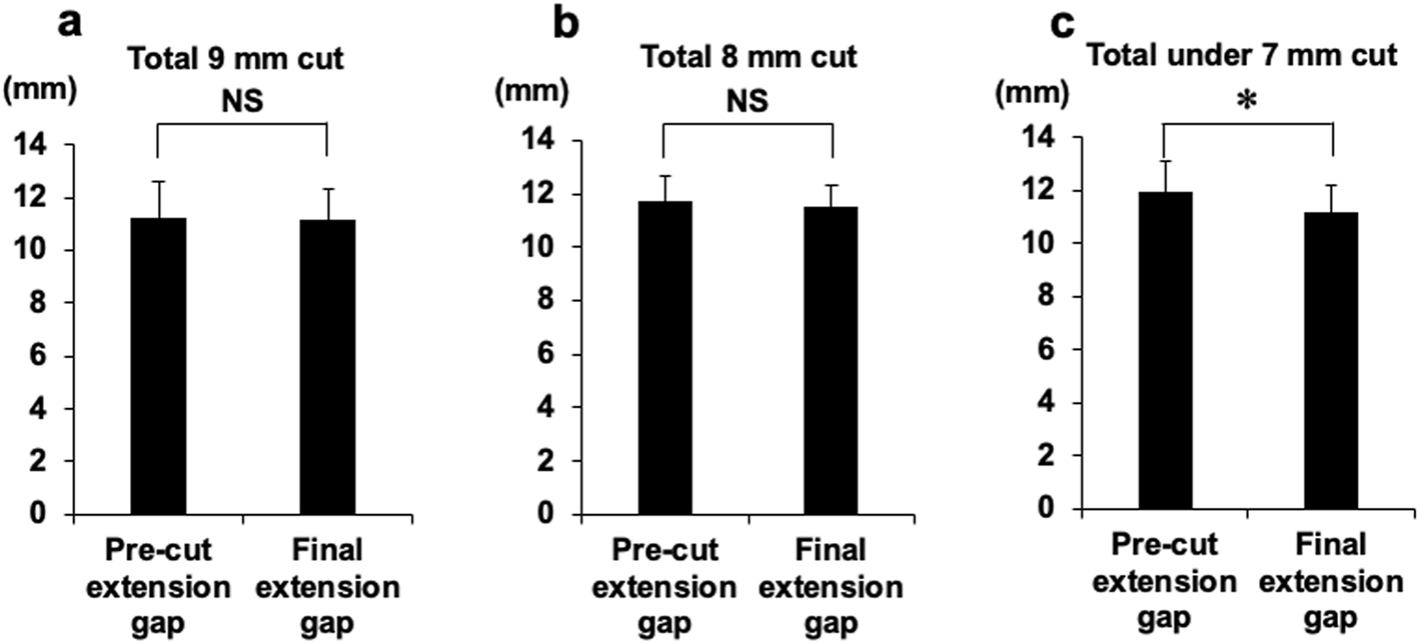
Relationship between gap control size and the final CGs at knee extension. The final CGs after gap control 0 mm posterior to the cutting level (total 9 mm cut) (**a**), the final CGs after gap control 1 mm posterior to the cutting level (total 8 mm cut) (**b**) and final CGs after gap control 2 and 3 mm posterior to the cutting level (total under 7 mm cut) (**c**) were compared with the CGs with PS PCT at knee extension. (*P < 0.01). CG, component gap; PS PCT, posterior-stabilised pre-cut trial component

Although the final CGs at knee extension were not statistically different from the CGs with PS PCT, we examined whether the gap control amount affected the final CGs at knee extension. The final CG groups of 0 mm (total 9-mm cut) and 1 mm (total 8-mm cut) posterior to the cutting level were not significantly different from the CGs with PS PCT (PS PCT vs. final CG at total 9-mm cut: 11.2 ± 1.4 vs. 11.1 ± 1.3 mm; PS PCT vs. final CG at total 8 mm cut: 11.7 ± 0.98 mm vs. 11.5 ± 0.88 mm) (Fig. 6a and b). Conversely, the final CG groups of 2 and 3 mm (total 7-mm and 8-mm cut, respectively) posterior to the cutting level were significantly decreased compared with that of CGs with PS PCT (PS PCT vs. final CG: 11.9 ± 1.2 vs. 11.1 ± 1.1 mm) (Fig. 6c).

### Clinical outcomes using pre-cut trial technique

**Fig. 7:**
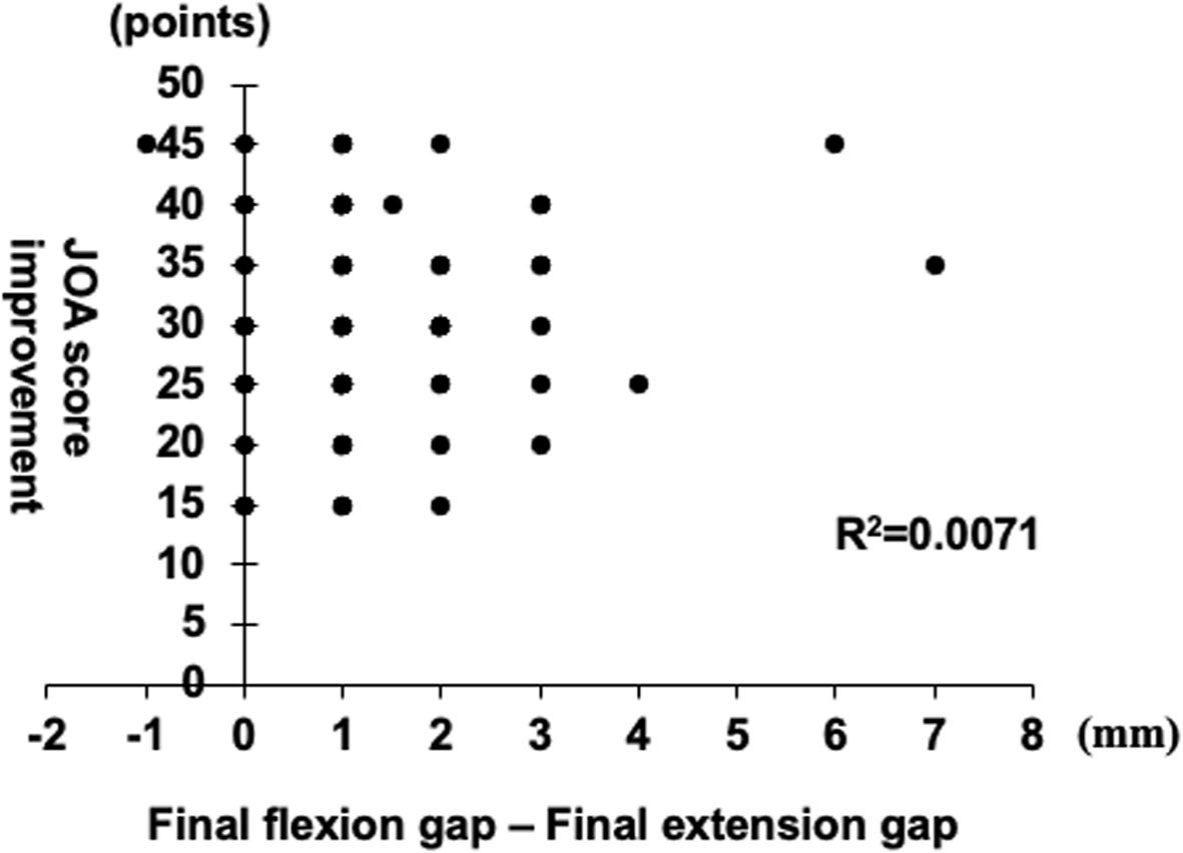
Clinical outcomes and gap. Relationship between the improvement of the clinical outcomes and gap differences (final flexion CG minus the final extension CG). CG, component gap

Pre- and postoperative JOA scores were 51.9 ± 8.2 (range, 35–75) and 82.9 ± 6.3 (range, 65–100) points, respectively. The postoperative JOA score significantly improved compared with the preoperative JOA score. Regression analysis showed that improvement from pre- to postoperative JOA scores (31.1 ± 8.4 points) was not significantly related to the gap difference (average 1.4 ± 1.3 mm; range, − 1–7 mm) between the final extension and flexion gaps (*R*^2^ = 0.0071, *P* = 0.49) (Fig. 7).

## Discussion

The most significant findings of the present study were that the CGs using PS PCT, both in extension and flexion, were not different from the final CGs set with trial femoral components. The final CGs were smaller than the estimated CGs obtained from the BGs without femoral components during knee extension. Moreover, we showed that the extension gaps increased by releasing the soft tissue around the intercondylar notch. Thus, the CGs obtained by PS PCT were the same as the final CGs in both knee extension and flexion. Moreover, the final CGs by the CG control using PS PCT were predictable before final bone resection.

The release of the femoral intercondylar notch capsule significantly increased the CG in only knee extension, compared with that before release. The difference between the CR- and PS-type femoral components is the presence of a cam structure in the PS femoral component. The cam occupies a large portion of the intercondylar notch, and the soft tissue around the intercondylar notch is subjected to tension. Okamoto et al. showed that 30 out of 54 knees in PS TKA required capsular release around the intercondylar notch to prevent flexion contracture, and noted that the extension gaps with the femoral component were dependent on the capsular tension around the cam [19]. In our study, release was not necessary in only one case, which met the exclusion criteria. The posterior protrusion of the cam in the femoral component may be the reason why most of our cases needed release; this is dependent on the shape of the femoral component, as determined by each manufacturer. The extension CGs made by PS PCT after release of the femoral intercondylar notch capsule were the same and were predictable as the final extension CGs with the trial femoral component. In contrast, the flexion CGs were unchanged.

Several studies have reported that the flexion gaps in TKA increased after resection of the PCL [6, 8, 17, 22]. In a pre-cut system, a 4-mm pre-cut surface on the lateral posterior femoral condyle was placed in the posterior part, (thickness: 4 mm) of the CR PCT [7]. However, in the pre-cut system of PS PCT, a 4-mm pre-cut surface on the femoral condyle was placed in the posterior part (thickness: 5 mm). The PS PCT has a 1-mm-thicker posterior part than the CR PCT; thus, the PS PCT fills an additional 1 mm of the gap in flexion compared with CR PCT (when not posterior of the cutting level). Our cases showed that an average of 1.1 mm posteriorly of cutting level was needed as flexion gap control; thus, a total of 2.1 mm was required (1.1 plus 1.0 mm) after pre-cutting to fill the flexion gap looseness compared with that of CR PCT (when not posterior of the cutting level). Therefore, gap control at 90° knee flexion is required during the PS TKA. The estimated final CGs obtained by PS PCT were not different from the final CGs with trial components after flexion gap control. These results suggest that release of the posterior capsule did not affect the CGs in flexion. Several studies demonstrated that the CGs were reduced for setting with trial femoral components only in extension, and were not affected in flexion [2, 16]. Our results support the notion that the final CGs with trial femoral components predicted the CGs, in both knee extension and flexion, using PS PCT.

We measured the BGs during extension and flexion using the OFR tensor. The CGs with PS PCT and final CGs were smaller than those estimated from BGs at knee extension only. Muratsu et al. showed that the CGs after placement trial femoral components of PS TKA (NexGen LPS Flex®, Zimmer Biomet Holdings) were significantly decreased by as much as 5.3 mm at knee extension only [16]. Minoda et al. showed that BGs in flexion tightened by over 1 mm compared with BGs in extension, and the BG differences between knee extension and flexion decreased; this suggested that the BGs in flexion should be made smaller than the BGs in extension before implantation, in order to minimise the mid-flexion laxity after implantation [15]. Our results suggest that final CGs with the trial femoral component can be estimated from BGs in knee flexion, but not in extension. In other words, when PS TKA using PS PCT is performed, surgeons are not required to estimate the final CGs from the BGs in both knee extension and flexion.

Further, we showed the relationship between the gap control amount and final CGs in knee extension. Although the final CGs at knee extension were not statistically different from the CGs with PS PCT, the final CGs with trial femoral components ≥ 2 mm posterior to the cutting level for flexion gap control were significantly smaller than those with PS PCT in the sub-analysis of extension CGs. Onodera et al. demonstrated that the posterior offset ratio depends on the shape of the femoral component, determined by each manufacturer, and the protrusion of the posterior condyle may cause knee flexion contracture due to the relative shortening of the posterior capsule [21]. Tsubosaka et al. confirmed that a larger posterior condylar offset reduced the CGs during knee extension, but not always in flexion [24]. Here, we quantitatively demonstrated that gap control of over 2 mm posterior to the cutting level (total under 7-mm cut) compared with the amount of femoral posterior condyle (total 9-mm cut) by the measured resection technique reduced the final CGs in knee extension. It is beneficial for surgeons to quantitatively estimate the final CGs before bone resection in knee extension and flexion.

The improvement of pre- to postoperative JOA scores was not related to the CG differences between the final extension and flexion gaps. The changes in ROM from pre- to post-surgery were not related to CG differences (data not shown). Although the influence of the difference between the flexion and extension gaps on the ROM and clinical outcome remains controversial [5, 13, 25], our results suggest that the average 1.4-mm gap difference between the extension and flexion gaps was not related to the clinical result.

This study has several limitations. First, the design of PS PCT is implant-specific and the results cannot be extended to all prosthesis designs. In detail, the posterior protrusion size of the cam in the femoral component depends on the component design and concept of each manufacturer. Although, the release of intercondylar notch of the posterior capsule was not needed in only one knee in the exclusion criteria, a previous report contrarily showed that 44% of knees required release of intercondylar condylar notch of the posterior capsule [19]. The influence of the cam on the extension gap should be considered when using PS TKA; however, it is not necessary in all cases. Analysis of the cam design for each femoral component is required in future studies. Second, the posterior reference guide for femoral resection is classified into three types according to the rotation centre for femoral component rotation, medial rotation, centre rotation and lateral rotation [14]. The pre-cut guide for initial femoral condylar resection is a posterior reference guide for the lateral rotation type for determining the femoral rotation [7]. The posterior reference guide for Persona® PS TKA using the measured resection technique is a centre rotation type. Thus, the resection was performed 9 mm from the femoral posterior centre, and the femoral component was set. Thus, the position of the PCT after pre-cutting of the femoral condyle is slightly different from the position of the femoral component after using the measured resection technique; however, we believe that a slight difference in the amount of femoral resection did not affect the final component position because the position of the femoral component was decided after the gap control in flexion. Third, the JOA score used as a clinical score in this study is commonly used in Japanese clinical practice [3, 18, 20]. The scoring system is an observer-based scoring system, and has already proven a significant correlation with other validated patient-reported outcomes [20]. However, we did not directly measure patient-reported outcomes, such as the Knee Injury and Osteoarthritis Outcome Score (KOOS) and Knee Society Score (KSS 2011, modified version), which are used internationally [9, 10]. The patient satisfaction scores in the KOOS and KSS 2011 may correlate with the final CG differences between extension and flexion.

## Conclusions

Extension CGs were affected by the cam structure in the PS femoral component and enlarged by releasing the capsule at the intercondylar notch. CGs estimated from the BGs were larger than the actual final CGs only in extension; therefore, surgeons should not estimate the final CGs in extension using BGs. The final CGs on knee extension and flexion during PS TKA did not differ from those obtained using PS PCT. The PS PCT technique could be used to estimate the gap lengths before final bone resection and could be a useful technique for CG control in PS TKA.

## Data Availability

The datasets generated and/or analysed during the current study are not publicly available due to compromised patient privacy but are available from the corresponding author on reasonable request.

## Abbreviations

BG: Bone gap
CG: Component gap
CR: Cruciate-retaining
FTA: Femorotibial angle
JOA: The Japanese Orthopaedic Association
KOOS: Knee Injury and Osteoarthritis Outcome Score
KSS: Knee Society Score
OA: Osteoarthritis
OFR: Offset Repo-Tensor®
PCL: Posterior cruciate ligament
PCT: Pre-cut trial component
PS: Posterior-stabilised
ROM: Range of motion
TKA: Total knee arthroplasty
